# Age-Related Decline in Disengaging Spatial Attention in Physiological Aging

**DOI:** 10.3390/brainsci15010006

**Published:** 2024-12-24

**Authors:** Tiziana Pedale, Serena Mastroberardino, Nicola Tambasco, Valerio Santangelo

**Affiliations:** 1 Department of Philosophy, Social Sciences & Education, University of Perugia, Piazza G. Ermini 1, 06123 Perugia, Italy; tiziana.pedale@unipg.it (T.P.); smastr@gmail.com (S.M.); 2 Functional Neuroimaging Laboratory, IRCCS Santa Lucia Foundation, Via Ardeatina 306, 00179 Rome, Italy; 3 Movement Disorders Center, Perugia General Hospital, University of Perugia, P.le Severi 1, 06132 Perugia, Italy; n.tambasco@libero.it

**Keywords:** alerting, orienting, conflict, executive function, attention, aging

## Abstract

**Background/Objectives:** Attention is a complex process involving various components such as alerting, orienting, and resolving conflicts. These components have been widely examined using the Attention Network Test (ANT), which has also been used to explore attentional decline associated with aging. However, discrepancies exist in the literature regarding which specific aspects of attention are most impacted by aging. These inconsistencies could be due to methodological issues such as group comparisons that may exaggerate differences between groups while flattening subtle variations within groups. **Methods:** To address this issue, we administered the ANT to 60 healthy participants aged between 62 and 90 years. Using a multivariate regression analysis, we examined whether increasing age was associated with changes in alerting, orienting, and conflict resolution, while controlling for overall performance in terms of both reaction times and accuracy. **Results:** The results showed a general and age-insensitive decline in two of the three attentional components: the alerting effect, which was abolished, and a large conflict effect, which was present regardless of age. In contrast, the orienting of spatial attention was found to linearly increase with increasing age. More focused analyses revealed that the ability to shift attention from the central (initial) to the peripheral (target) location slowed down as a function of age. **Conclusions:** These results suggest that aging is associated with a greater difficulty in disengaging endogenous attention from the central, uninformative cue to direct attention on task-relevant peripheral targets.

## 1. Introduction

Attention is a fundamental and multifaceted process that supports a variety of high-level cognitive functions, from stimulus to response selection, from conflict and distraction avoidance to control of task performance (see, for reviews, [1,2,3]). A widely recognized and influential model has been proposed whereby attention operates through three key components: alerting, which refers to the ability to respond to high-intensity stimuli that alter one’s state of arousal; orienting, which pertains to the ability to focus attention selectively on a particular stimulus or location; and executive functioning, which involves the ability to regulate inhibitory control and resolve conflicts [4]. These different components have been extensively investigated using the Attention Network Test (ANT), originally developed by Fan and colleagues [5]. The ANT is a computerized, reaction time (RT)-based test, which consists of the discrimination of the target stimulus that can be flanked by congruent, incongruent, or neutral stimuli. The spatial location where the target appears can be anticipated by a spatial (i.e., peripheral) cue, while in some trials a spatially unpredictive (i.e., central or double) cue or no cue at all is presented. The comparison of the participant’s performance between trials with no cues and double cues provides a measure of alerting, while the comparison between trials with a central cue and peripheral cues provides a measure of orienting. Finally, the comparison between trials with incongruent and congruent targets provides a measure of executive functioning.

The ANT has been widely used to characterize alerting, orienting, and executive functions in various populations, including healthy individuals and patients with attention-related disorders (for a recent meta-analysis, see [6]; see also [7,8]). The ANT has also been utilized to examine the development of and decline in alerting, orienting, and executive functions across the lifespan [9,10,11]. In the context of physiological aging, while general attentional difficulties in attentional and working memory tasks are well-documented [12,13,14], the literature shows inconsistencies regarding the specific functioning of the three components assessed by the ANT [9,15], leading to ongoing debate. Regarding the alerting component, there is relatively high consistency across studies reporting an age-related decline in alertness mechanisms [11,16,17,18,19,20,21,22,23], although opposite patterns have also been observed in a few studies [24,25]. Casagrande and colleagues [24] found no differences in alerting scores between younger and older adults when using a modified version of the ANT. This finding, however, may be attributed to the nature of the alerting stimulus in their study, which was auditory rather than visual, potentially alerting participants in a more automatic manner [26]. In contrast, Fernandez-Duque and colleagues [25] reported higher orientation scores in older adults compared to younger participants, suggesting that an alerting signal may be particularly advantageous for older individuals. Findings regarding executive function are also inconsistent. The current literature reports both age-related increases in conflict scores [23,27,28,29], suggesting a decline in executive control due to greater interference from incongruent stimuli, and age-invariant scores in conflict control, indicating equivalent efficiency of the executive component from adulthood to old age [17,18,19,20,22]. Similarly, for the orienting effect, an fMRI study using the ANT reported lower orienting scores in older adults compared to younger adults [16], showing a reduced benefit in the presence of a spatial cue. However, other studies have reported age-invariant scores [18,23,25,28], or even higher orienting scores with age ([11,30]; see also [31] for a selectively increased orienting score in a modified version of the ANT using faces).

While this variety of findings could stem from subtle experimental differences across the studies mentioned above (e.g., variations in stimulus size, brightness, duration, or whether general behavioral performance, such as reaction times and accuracy, is controlled for), we propose a methodological caveat. Specifically, the investigation of attentional decline in physiological aging often relies on comparisons between groups of different ages (e.g., older adults vs. younger adults). This approach might exaggerate differences that are minimal between groups or, conversely, flatten existing within-group differences. This idea is supported by a recent meta-analysis by Klein and colleagues [9]. Indeed, while a consistent decline in the alerting effect with aging has been revealed, more puzzling findings have appeared regarding the orienting and executive components. Specifically, regarding the orienting function, Klein et al. found that when comparing groups spanning from young adulthood to old age, orienting scores did not appear to be significantly modulated by age, showing only a trend toward increased orienting scores in older participants. However, they also observed that when the effect of age on orienting scores was specifically tested within older adults (aged 65–87), there was a significant decrease in orienting scores as a function of age (see also Verissimo et al. [11], for opposite findings for the orienting score). Klein and colleagues [9] reported a similar pattern for executive scores. When considering a larger lifespan (younger and older adults), a robust effect of aging (i.e., an increase in executive/conflict scores) was observed. However, within a narrower older age range (65–87 years), there was a trend toward decreased executive/conflict scores with increasing age (see also Verissimo et al. [11], who reported a consistent result).

Taken together, these findings suggest that inconsistencies in the literature may be heavily influenced by the choice of control groups. This highlights the fact that the effects of aging on alerting, orienting, and executive functions might be better studied within a homogeneous sample, such as older adults exclusively. One recent study has specifically investigated alerting, orienting, and executive functions in a homogeneous densely sampled group of older adults: the study by Verissimo and colleagues [11]. However, this study reported results that diverge from the broader literature, including small alerting, orienting, and conflict effects, as well as an opposite pattern for the orienting effect (i.e., increased amplitude with age), as noted by Klein and colleagues in their meta-analysis [9]. These observations underscore the need for further investigation of the effects of age on attention components in aging, with a specific focus on a narrower age range.

Here, we aimed to contribute to this ongoing debate by using a multivariate linear regression model to investigate whether increasing age predicts alerting, orienting, and executive function scores in a group of sixty healthy older participants (mean age: 76.1 years; range: 62–90 years), while controlling for the participants’ general behavioral performance. As an additional constraint not previously implemented in the extant literature, here we estimated the effect of aging on each of the attentional and behavioral scores simultaneously, that is, while controlling for each of the dependent variables included in the multivariate model (for a similar approach, see [32]). Based on the current literature [9,11,15], we might anticipate an age-related decline in alerting scores and an age-related increase in conflict scores. In contrast, the trajectory of orienting scores remains less clear, as both an increase and a decrease in this score could be hypothesized (see e.g., [9,11]).

## 2. Materials and Methods

### 2.1. Participants

A total of 60 healthy participants (31 males, 29 females; mean age ± standard deviation = 76.1 ± 6.8 years; range = 62–90 years) volunteered for and took part in the study. They were recruited across three Italian recreational centers for elderly people: “Associazione AUSER”, Foligno, Perugia; “Associazione socio-culturale Ferro di Cavallo”, Perugia; “Centro Sociale Anziani Cittaducale”, Cittaducale, Rieti. The exclusion criteria encompassed the presence of any self-reported neurological, psychiatric, or cognitive disorder (including subjective memory decline), as well as being under pharmacological treatment for such conditions, including the use of psychoactive medications like antidepressants and anxiolytics. All of the participants had normal or corrected-to-normal vision and were naïve as to the main purpose of the study, which was conducted in accordance with the research ethics principles of the Declaration of Helsinki. All the participants provided informed consent, and their privacy rights have been observed. The sample size was a priori estimated for our main analysis (a multiple regression), taking into account a medium effect size (f2) of 0.15, a statistical power of 0.80, a significance level of 0.05, and one predictor (i.e., the participants’ age). This calculation resulted in a minimum sample size of 54 participants. The study was performed in compliance with relevant laws and institutional guidelines and all procedures have been approved by the independent Ethics Committee of Umbria (CER; name of the project: PD_QUIP_01; reference number: 3194/19; date of approval: 14 February 2018); the study was conducted as part of the gathering normative data on the neurocognitive profile in Parkinson’s disease to better understand how the disease affects cognitive functions such as memory, attention, and decision making.

### 2.2. Stimuli and Task

Participants sat in a quiet room in front of a laptop computer. The laptop display was placed approximately 50 cm from the viewer (display size = 29° × 22° of visual angle). Participants were administered with the Attention Network Test (ANT; [5]), running on PsychoPy 2022.2.5 (https://www.psychopy.org (accessed on 3 April 2023)).

**Figure 1:**
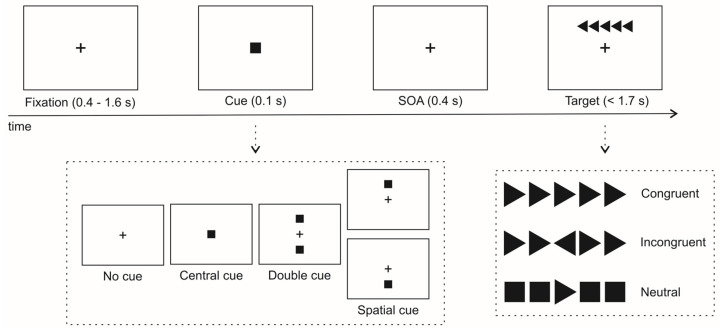
Schematic diagram showing the sequence of events during a sample ANT trial. After an initial fixation cross, a central, double, or spatial cue (or no cue) was presented. After the stimulus onset asynchrony (SOA), a target was presented, consisting of a central arrow oriented either to the left or to the right. The target arrow could be either presented together with neutral squares (neutral trials) or together with arrows oriented in the same or opposite direction (congruent and incongruent trials, respectively).

Figure 1 shows the stimuli and an example trial. Each trial began with the presentation of a black fixation cross (on a grey background) for a duration between 400 and 1600 ms. Following this, an attentional cue could be shown for 100 ms. The cue could either replace the fixation point (i.e., “central cue” condition), appear both above and below the fixation point (i.e., “double cue” condition), or be placed either above or below the fixation point (i.e., “spatial cue” condition). In some trials, no cue was presented (i.e., “no cue” condition). After a brief 400 ms fixation period, the target appeared. This consisted of a black triangle-arrow displayed either above or below the central fixation point, vertically aligned with the center of the screen. The triangle arrow could be oriented to the left or right. The target could be presented with black squares (“neutral” trials) or with flanking triangle arrows pointing in the same direction (“congruent” trials) or the opposite direction (“incongruent” trials). Participants were instructed to discriminate the target’s left vs. right orientation by pressing one of two response keys as quickly as possible within a 1700 ms time window.

The experiment was divided into three blocks, each containing 96 trials. The trials were created by combining four cue conditions (no cue, central cue, double cue, and spatial cue), two target locations (above vs. below fixation), two target orientations (right vs. left), three target types (congruent, incongruent, and neutral), and two repetitions. Before starting the task, participants completed a practice session of 24 trials, during which they received feedback on their performance.

### 2.3. Data Analysis

To account for general task performance, reaction times (RTs), including only correct trials, and the percentage of accuracy were computed across all trial types. Moreover, we calculated the overall task accuracy (mean ± SD, 84.2 ± 17.3%), and participants with an accuracy rate lower than two standard deviations below the group mean were excluded from further analyses. This criterion led to the exclusion of two participants, leading to a final sample of 58 participants (31 males, 27 females; mean age = 76.1 ± 6.9 years; range = 62–90 years; overall task accuracy = 86.2 ± 13.3%). Next, we computed three specific measures related to the ANT: alerting, orienting, and conflict (cf. [5]). To do this, we first calculated the participant’s mean reaction times (RTs) for each cue condition (across the three target types) and for each target type (across the four cue conditions). Alerting was computed by subtracting mean RTs for “no cue” − mean RTs for “double cue” trials; orienting was computed by subtracting mean RTs for “central cue” − mean RTs for “spatial cue” trials; finally, conflict was computed by subtracting mean RTs for “incongruent” − mean RTs for “congruent” trials.

To assess the effectiveness of the alerting, orienting, and conflict components of attention in our sample, we performed one-tailed one-sample *t*-tests (both frequentist and Bayesian) to determine whether the obtained scores were significantly greater than 0. Bayesian analyses were conducted to evaluate the strength of evidence for each attention component, following the criteria set by Keysers and colleagues [33]: BF^10^ < 1 (no evidence), BF^10^ between 1 and 3 (anecdotal evidence), BF^10^ between 3 and 10 (substantial evidence), BF^10^ between 10 and 30 (strong evidence), BF^10^ between 30 and 100 (very strong evidence), and BF^10^ > 100 (decisive evidence).

To investigate the effect of aging on attention (i.e., alerting, orienting, and conflict), we employed a multivariate regression model (MRM). MRM is a robust tool for modeling the linear relationship between a single independent variable (predictor) and multiple dependent variables simultaneously. Using MRM, rather than separate regression models, allows us to estimate the specific influence of the predictor on the dependent variables while controlling for the variability of each dependent variable (see [32] for a similar approach). The current MRM included age as a predictor and alerting, orienting and conflict as dependent variables. The variables related to the general task performance including all trial types (i.e., RTs and percentage of accuracy) were also included in the model as dependent variables to take account of this variability, thus ruling out any potentially confounding explanations in our main results due to the participants’ general task performance. The attentional components that resulted to be significantly predicted by age were further explored using a repeated measures analysis of covariance (ANCOVA) with the age as a continuous covariate and the two trial types used to compute each attentional score as two levels repeated measure factor: i.e., “no cue” vs. “double cue” for alerting; “central cue” vs. “spatial cue” for orienting; “incongruent” vs. “congruent” target for conflict. This approach enabled us to clarify the specific contribution of age on the two trial types used to compute the attentional score.

## 3. Results

Preliminarily, a two-way repeated-measures analysis of variance (ANOVA) was conducted on the RT and accuracy data with the factors cue type (no cue, central cue, double cue, spatial cue) and target type (congruent, neutral, incongruent) (see Table 1). A Greenhouse–Geisser adjustment was applied to correct for violations of the sphericity assumption.

Both the ANOVAs on the RTs and accuracy revealed a main effect of target type (RTs: [F(1.3, 72.5) = 173, *p* < 0.001, η^2^ = 0.752]; accuracy: [F(1.1, 62.1) = 51.3, *p* < 0.001, η^2^ = 0.474]), indicating a decrease in performance following incongruent (mean RT = 899 ms; mean accuracy = 76.1%) compared to congruent (747 ms; 91.3%) or neutral (762 ms; 91.2%) targets. The ANOVA on the RTs also revealed a main effect of cue type, [F(2.5, 141.4) = 8.9, *p* < 0.001, η^2^ = 0.134], indicating faster responses following spatial cues (783 ms) than following no cues (812 ms), central cues (807 ms) or double cues (809 ms) (while the main effect of cue type was not significant for the accuracy data: [F(3, 171) < 1, n.s.]). None of the ANOVAs showed significant interactions between cue type and target type (RTs: [F(3.6, 207.2) = 1.1, n.s.]; accuracy: [F(4.8, 274.7) = 2.1, *p* = 0.066, η^2^ = 0.036]).

Then, we moved on to analyzing alerting, orienting, and conflict effects in our sample. For the alerting effect, we observed a small average effect of 6 ± 5 ms (mean ± standard error). As this effect was computed by subtracting mean RTs for “no cue” − mean RTs for “double cue” trials, overall our participants did not take advantage of the “alerting” double cue signal, showing similar performance in trials with no cues or double cues [t(57) = 1.04, *p =* 0.15, Cohen’s d = 0.14; BF^10^ = 0.24]. For the orienting effect, we found an average of 25 ± 5 ms, indicating decisive evidence for overall faster responses following spatial cues than central cues [t(57) = 5.02, *p* < 0.001, Cohen’s d = 0.66; BF^10^ > 100]. Finally, for the conflict effect, we found an average of 153 ± 11 ms, indicating again decisive evidence for slower responses following incongruent than congruent trials [t(57) = 13.51, *p* < 0.001, Cohen’s d = 1.77; BF^10^ > 100].

**Figure 2:**
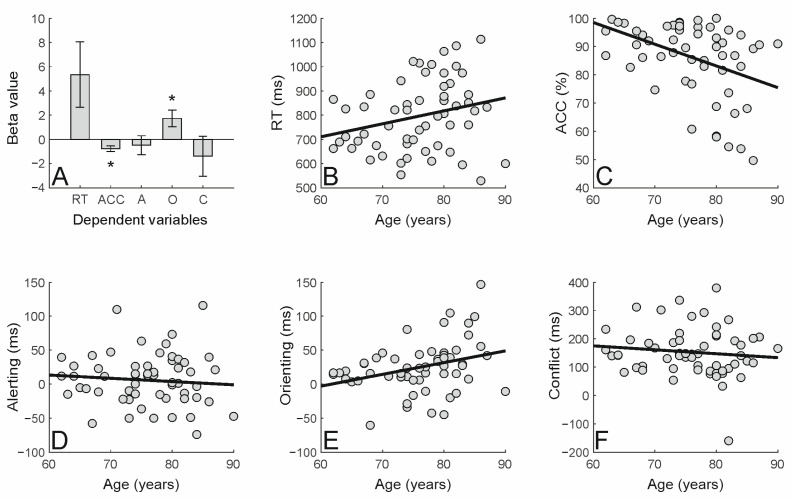
(**A**) Standardized regression coefficients (beta values) derived from the multivariate regression model (MRM) used to assess the predictivity of age on the measured variables: Reaction times (RT), percentage of accuracy (ACC), alerting (A), orienting (O), and conflict (C; see also Table 2; the error bars represent the standard error of the mean). The asterisks indicate significant effects (*p* < 0.05) (**B**–**F**) Scatter plots illustrating the relationship between the measured variables and the participants’ age.

Then, in line with the main aim of the present study, we examined the impact of increased participants’ age on attention-related variables (i.e., alerting, orienting, and conflict) and general task performance (RTs and accuracy) using MRM (see Table 2). The MRM revealed that the increase in participants’ age significantly predicted the participants’ overall performance on the ANT in terms of percentage of accuracy (*p* = 0.002; see Figure 2A, bar 2), and with a trend in terms of RTs (*p* = 0.053; see Figure 2A, bar 1). This indicates that the older the participants’ age, the higher the RTs (see Figure 2B) and the lower the accuracy (see Figure 2C). Crucially, however, the increase in the participants’ age only modulated attentional orienting (*p* = 0.014; see Figure 2A, bar 4 and Figure 2E), which was found to increase significantly as a function of age. Conversely, participants’ age did not predict alerting (*p* = 0.541; see Figure 2A,D) or conflict (*p* = 0.401; see Figure 2A,F) effects.

**Figure 3:**
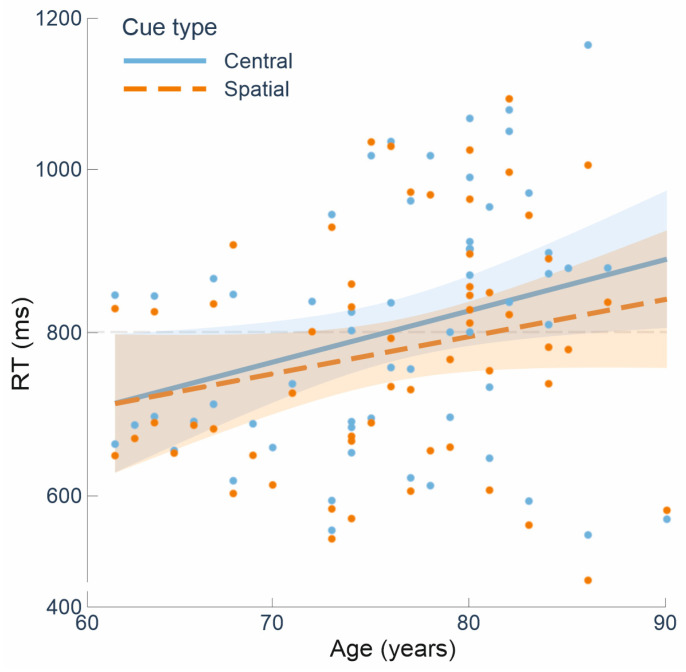
Scatter plot illustrating the relationship between the reaction times (RT) to central and spatial cues and the participants’ age. The bands around the regression lines represent the 95% confidence interval.

The increase in the orienting effect as a function of age was further investigated using an ANCOVA model. The orienting index was computed by subtracting the mean RTs following central cues minus the mean RTs following spatial cues. Accordingly, the increased orienting score as a function of age could be due to either age-related changes in the RTs following the uninformative central cues, or the spatially informative cues. To untangle this issue, we performed an ANCOVA on the mean RTs data, with the cue type as a repeated-measure factor (two levels: “central cue” and “spatial cue”), and age as a continuous predictor. The ANCOVA revealed a main effect of cue type [F(1, 56) = 4.19, *p* = 0.045, η^2^ = 0.070], indicating slower responses following central cues (mean RT = 801 ms) than spatial cues (777 ms). However, this model revealed only a marginal effect of age [F(1, 56) = 4.02, *p* = 0.05, η^2^ = 0.067], which was further clarified by a significant interaction between cue type and age [F(1, 56) = 6.40, *p* = 0.014, η^2^ = 0.103]. The interaction indicates that the effect of age on the orienting score was driven by a greater slowing of RTs as a function of age following the uninformative “central cue”, as compared to the “spatial cue” (compare in Figure 3 the steepness of the regression lines as a function of age for the central vs. spatial cues).

## 4. Discussion

The present study examined age-related differences in older adults using the ANT. Consistent with the literature, we observed a general decline in performance (i.e., slower reaction times and lower accuracy rates) in our sample of elderly participants following incongruent targets compared to congruent or neutral targets. Moreover, faster responses were observed following the presentation of spatially informative cues compared to spatially uninformative cues and the no cue condition. Similarly, we found attentional effects comparable to those already described in the extant literature, namely, a reduced alerting effect [11,16,17,18,19,20,21,22,23,34,35], and a large conflict [23,27,28,29] and orienting effect [11,30,31].

With respect to alerting, the Bayesian analysis conclusively revealed an abolished alerting effect in our sample, indicating that our participants were unable to benefit from the alerting cues compared to the no cue condition. Consistently, the MRM showed no modulation in the magnitude of the alerting effect as a function of participant age. The reduction/elimination of the alerting effect with aging is in good agreement with the previous literature, either when the alerting mechanisms were tested alone [35] or together with orienting and conflict within the ANT paradigm, as in the present study [17,18,19,21,22,23]. Here, we extended this notion by showing that the decrease/elimination of the alerting effect may be evident as early as 62 years of age and does not appear to be further modulated by physiological aging.

On the contrary, the Bayesian analyses provided decisive evidence for the existence of both conflict and orienting effects in our sample of participants. However, among these two attentional components, only the orienting score was found to increase as a function of participants’ age, while the conflict effect (due to slower responses following incongruent than congruent trials) was shown to be stable from 62 to 90 years of age. This latter evidence suggests a comparable difficulty in inhibiting task-irrelevant incongruent information in our sample of older participants, regardless of age [13,36,37]. This notion is in line with most of the existing literature showing a decline in executive control in aging, as demonstrated by studies using the ANT [23,27,28,29], but also with different paradigms, such as task switching [38,39] or stimulus inhibition [13,36,40,41] (but see also the review by McDonough and colleagues [15], for studies suggesting a delayed decline of attentional control in aging, and [11] for a study showing even a reduced executive score as a function of age). Although it may seem surprising that no worsening of the conflict score emerged in our sample after the age of 62, this can be explained by the fact that our model accounted for the general decline in both accuracy and reaction times. Once this overall age-related decline was controlled for, the difficulty in filtering out irrelevant stimuli was found to remain stable from 62 to 90 years of age.

With respect to the orienting effect in aging, more heterogeneous results have been reported in the literature (see [18,23,25,28,42,43,44] for studies showing no age modulation of attentional orienting; see [11,30,31,45,46,47,48] for studies revealing age-related differences in orienting effect; for a review, see [49]). Recently, a developmental trajectory in the orienting of spatial attention in aging was reported by a study that found an increased amplitude of orienting effects in older participants when using cues representing socially relevant stimuli (i.e., faces) in a modified social version of the ANT [31]. This effect was interpreted in support of an orienting bias to social stimuli with aging, whereas a similar age-related modulation was not observed when using the traditional version of the ANT. Enhanced orienting effects have also been reported in physiological aging by other studies, indicating a greater and more persistent influence of peripheral non-predictive cues in older compared to younger adults [46,47,48], suggesting enhanced involuntary orienting mechanisms. Furthermore, our results are consistent with those of Verissimo and colleagues [11], who found increased orienting scores as a function of age in a densely sampled older population. Here, we shed new light on this issue revealing decisive evidence—with the Bayesian approach—of an orienting effect in our sample of older participants. Furthermore, we demonstrated a specific sensitivity of this effect as a function of participant age, showing a linear increase in the magnitude of the orienting effect as a function of age in our sample of older participants.

The orienting of spatial attention requires disengagement from the currently attended location in order to shift and engage attention to a novel location [50]. Accordingly, the current age-related increase in the magnitude of the orienting effect could be due to two alternative mechanisms: i.e., increased orienting benefits in the presence of the spatially predictive cues (as suggested by Verissimo and colleagues [11]) vs. increased costs of disengagement from the uninformative central cues. Here, we found that the current age-related orienting effect is mainly driven by the latter alternative, namely a greater slowness in direct attention to the target when it is preceded by the uninformative central cues as compared to spatial cues (cf. Figure 3). This finding suggests that the cost of disengaging endogenous attention from the uninformative central cue to shift to the unknown target location increases significantly with age, as compared to the condition in which spatial cues correctly predict the target position (see also [13,51,52] for consistent results showing age-related reorienting costs). Although this study was not specifically designed to distinguish between these two mechanisms (orienting benefits vs. disengagement costs), this finding casts doubt on the interpretation that aging is associated with improved orienting mechanisms [11].

There are, however, some limitations that should be taken into account in future research. We did not perform a neuropsychological assessment of the participants to rule out the absence of cognitive impairments beyond self-report. While self-report measures provided an initial safeguard, the lack of standardized neuropsychological tests leaves some uncertainty regarding the presence of undiagnosed or subtle cognitive deficits within our sample. In addition, we did not collect information on the education level of our participants, which may be an important factor to consider in future research in this area, nor on their physical exercise performances [53,54], which could potentially affect reflex readiness. As a future direction, working memory training could be explored for older participants, given its positive effects on ANT performance and reaction times [55], potentially mitigating the increased difficulty in orienting with age.

## 5. Conclusions

In the current study, we provided evidence for a general decline in attentional components with age. Specifically, we observed a general and age-insensitive decline in two of the three attentional components measured by the ANT: the alerting effect was eliminated and a large conflict effect was present regardless of age. In contrast, only the orienting score was specifically sensitive to increasing participant age. More focused analyses revealed that this increase was driven by greater difficulty in shifting attention from the central (initial) location to the peripheral (target) location with age. These findings suggest that aging is associated with increased difficulty in directing endogenous attention toward relevant locations.

## Figures and Tables

**Table 1:** Mean reaction times, RTs (±standard error), and mean percentage of accuracy, ACC (±standard error), for the different cue and target conditions.

	Congruent		Neutral		Incongruent	
	RT (ms)	ACC (%)	RT (ms)	ACC (%)	RT (ms)	ACC (%)
No cue	754 ± 20	91.5 ± 1.3	778 ± 20	90.3 ± 1.5	903 ± 22	77.7 ± 3.0
Central cue	757 ± 21	91.2 ± 1.3	760 ± 20	90.7 ± 1.3	904 ± 22	76.2 ± 3.0
Double cue	745 ± 20	90.9 ± 1.5	770 ± 20	92.0 ± 1.3	913 ± 23	75.7 ± 3.3
Spatial cue	733 ± 19	91.5 ± 1.2	741 ± 20	91.6 ± 1.4	874 ± 24	74.6 ± 3.2

**Table 2:** Standardized regression coefficients (betas), *t*-values, significance (*p*), 95% confidence interval (C.I., lower, upper boundaries), and effect size (η^2^) resulted from the multivariate regression model (MRM), showing the impact of the predictor (Age) on the reaction times (RTs), percentage of accuracy (ACC) and orienting dependent variables.

Predictor	Dependent	Beta	*t*-Value	*p*	95% C.I.	η^2^
Age	RTs	5.351	1.979	0.053	−0.066, 10.768	0.065
	ACC	−0.768	−3.278	0.002	−1.238, −0.299	0.161
	Alerting	−0.478	−0.615	0.541	−2.034, 1.078	0.007
	Orienting	1.732	2.530	0.014	0.361, 3.103	0.103
	Conflict	−1.394	−0.847	0.401	−4.691, 1.904	0.013

## Data Availability

The original data presented in the study are openly available in Mendeley Data at https://doi.org/10.17632/f7hydbkgw4.1.
